# Belantamab mafodotin in triple‐refractory multiple myeloma patients: A retro‐prospective observational study in Italy

**DOI:** 10.1002/jha2.907

**Published:** 2024-04-30

**Authors:** Francesca Fazio, Maria Teresa Petrucci, Laura Corvatta, Alfonso Piciocchi, Roberta Della Pepa, Paola Tacchetti, Maurizio Musso, Renato Zambello, Angelo Belotti, Sara Bringhen, Elisabetta Antonioli, Concetta Conticello, Nicola Di Renzo, Valerio De Stefano, Pellegrino Musto, Barbara Gamberi, Daniele Derudas, Mario Boccadoro, Massimo Offidani, Sonia Morè

**Affiliations:** ^1^ Hematology Unit, Department of Translational and Precision Medicine Azienda Ospedaliera Policlinico Umberto I Sapienza University of Rome Rome Italy; ^2^ Unità Operativa Complessa di Medicina Ospedale Profili Fabriano Italy; ^3^ GIMEMA Foundation Rome Italy; ^4^ Hematology Unit, Department of Clinical Medicine and Surgery University of Naples “Federico II” Naples Italy; ^5^ IRCCS Azienda Ospedaliero‐Universitaria di Bologna‐Istituto di Ematologia “Seràgnoli” Bologna Italy; ^6^ Oncoematology and BMT Unit, Oncology Department Ospedale La maddalena Palermo Palermo Italy; ^7^ Hematology Unit Department of Medicine University of Padova Padua Italy; ^8^ Department of Hematology ASST Spedali Civili di Brescia Brescia Italy; ^9^ SSD Clinical Trial in Oncoematologia e Mieloma Multiplo Department of Oncology Azienda Ospedaliero‐Universitaria Città della Salute e della Scienza di Torino Turin Italy; ^10^ Haematology Unit Careggi University Hospital Florence Italy; ^11^ Division of Haematology and BMT A.O.U. ‘Policlinico‐San Marco’ Catania Italy; ^12^ Hematology and Stem Cell Transplant Unit “Vito Fazzi” Hospital Lecce Italy; ^13^ Section of Hematology Department of Radiological and Hematological Sciences Catholic University Fondazione Policlinico A Gemelli IRCCS Rome Italy; ^14^ Department of Precision and Regenerative Medicine and Ionian Area “Aldo Moro” University School of Medicine, and Unit of Hematology and Stem Cell Transplantation AOUC Policlinico Bari Italy; ^15^ Hematology Unit Azienda USL‐ IRCCS di Reggio Emilia Reggio Emilia Italy; ^16^ SC di Ematologia e CTMO ‐ Oncologico Oncologico di Riferimento Regionale “A. Businco” ‐ ARNAS “G. Brotzu” Cagliari Italy; ^17^ European Myeloma Network Turin Italy; ^18^ Department of Hematology Azienda Ospedaliero Universitaria delle Marche Ancona Italy

**Keywords:** belantamab mafodotin, multiple myeloma, real‐world

## Abstract

Belantamab mafodotin is the first‐in‐class antibody‐drug conjugates targeting B‐cell maturation antigen to have demonstrated effectiveness in triple‐class refractory multiple myeloma (TCR‐MM) patients. We performed a retrospective study including 78 TCR patients, with at least four prior lines of therapy (LOTs), who received belantamab mafodotin within named patient program and expanded access program in Italy between 2020 and 2022. Median age was 65 years (range 42–86 years), ECOG performance status was ≥1 in 45% of patients. Overall, a clinical benefit was obtained in 36 out of 74 evaluable patients (49%), with 43%, 28%, and 13.5% achieving at least partial response, very good partial response, and complete response, respectively. After a median follow‐up of 12 months (range 6–21 months), median duration of response, progression‐free survival (PFS), and overall survival (OS) were 14, 5.5, and 12 months, respectively. Age >70 years, good performance status and response were associated with longer PFS and OS. Keratopathy occurred in 58% of patients (G3 2.5%), corneal symptoms in 32% (G3 1.2%) and a reduction in visual acuity in 14%. Grade 3 thrombocytopenia occurred in 9% of patients. Only 3% of patients discontinued belantamab mafodotin because of side effects. This real‐life study demonstrated significant and durable responses of belantamab in TCR‐MM patients with four prior LOTs, otherwise ineligible for novel immunotherapies.

## 1 INTRODUCTION

The growing use of combinations including immunomodulatory (IMiDs) agents, proteasome inhibitors (PI) and monoclonal antibody is generating an increasingly large population of patients who can become triple‐class refractory. These patients represent a serious unmet clinical need because their prognosis is very dismal [1, 2] and no effective therapeutic options are available. Indeed, access to new immunotherapies as CAR‐T cells or bispecifics is limited due to the need for hospitalization or complex logistics, poor performance status—as generally seen in advanced phases of the disease—and finally the high cost. Belantamab mafodotin is the first‐in‐class antibody‐drug conjugates (ADC) targeting B‐cell maturation antigen (BCMA) approved for relapsed/refractory multiple myeloma (RRMM) who received at least four prior LOTs including a PI, an IMiD, and an anti‐CD38 mAb. Approval was based on the results of the phase II, open‐label, two‐arm, multicenter DREAMM‐2 trial [3]—primary endpoint was overall response rate (ORR). RRMM patients with a median of six prior LOTs were allocated to receive belantamab mafodotin 2.5 or 3.4 mg/kg every 3 weeks until disease progression or unacceptable toxicity. In the final analysis of that study, ORR was 32% and 35%, median duration of response was 12.5 and 6.2 months, and median progression‐free survival (PFS) was 2.8 and 3.9 months in the 2.5 and 3.4 cohort, respectively. Median overall survival (OS) was 15.3 months for 2.5 mg/kg and 14 months for 3.4 mg/kg; in patients achieving at least very good partial response (VGPR), median OS was 30.7 and 35.5 months, respectively. Ocular toxicity, mainly keratopathy, was found to be a peculiar adverse event of belantamab mafodotin, occurring in 71% of patients (grade ≥3 = 29%) receiving of 2.5 mg/kg, the approved dosage. Only 3% of patients in both arms permanently discontinued treatment due to ocular toxicities. Recently several studies on belantamab mafodotin as monotherapy in the real‐life context have been published, showing an ORR ranging from 27% to 52% [4, 5, 6, 7, 8, 9, 10, 11, 12, 13]. Results of these studies are summarized in Table 1. In this study, we evaluated efficacy and safety of belantamab mafodotin in real‐life RRMM patients in Italy.

**TABLE 1:** Real life studies of belantamab mafodotin.

Title	Patients (n)	Population: median of prior lines of therapy (range), median age (range)	Outcomes (ORR, mPFS, mDOR)	Safety: keratopathy grade ≥3 (%)
USA experiences				
“Real‐life” data of the efficacy and safety of belantamab mafodotin in relapsed multiple myeloma—the Mayo Clinic experience [4]	36	8 (7–11) 61 (37–83)	33 2 14.3	8
Impact of belantamab mafodotin‐induced ocular toxicity on outcomes of patients with advanced multiple myeloma [8]	38	8 (2–15) 67 (49–90)	–	14
Retrospective, single‐center, real‐world experience of belantamab mafodotin in relapsed/refractory multiple myeloma [7]	39	7 (3–16) 66 (39–89)	32 2.8 11	12
Belantamab mafodotin in patients with relapsed/refractory multiple myeloma, a real‐world single center experience [14]	90	6 (2–14) 68 (37–88)	42 4 13.1	16
Belantamab mafodotin (Belamaf) for relapsed/refractory multiple myeloma (RRMM): a real‐world observational study [15]	137	5 (4–7) 68 (±10)	30.2 5.4 –	38.6
Asian experiences				
Real‐world experience with belantamab mafodotin therapy for relapsed/ refractory multiple myeloma: a multicenter retrospective study [16]	106	6 (2–11) 69 (36–88)	45.5 4.7 8.1	24
European experiences				
Belantamab mafodotin in patients with relapsed and refractory multiple myeloma who have received at least one PI, one IMID and one anti‐cd38 mAb: a retro‐prospective Italian observational study [17]	67	5 66 (42–82)	31 3.7 13.8	13
Efficacy and safety of belantamab‐mafodotin in triplerefractory multiple myeloma patients: a multicentric real‐life experience [11]	28	6 (3–14) 67.5 (51–83)	40 3 –	11
Belantamab mafodotinin patients with relapsed/refractory multiple myeloma included in the compassionate use or the expanded access program. Experience with a Spanish cohort [18]	156	5 (4–6) 72.5 (64–77)	46.4 3.6 13.9	17.9
Effectiveness and safety of belantamab mafodotin in patients with relapsed or refractory multiple myeloma in real‐life setting: The ALFA study [19]	184	5 70 (63–76)	32.7 2.4 –	8.2
Real‐world study of the efficacy and safety of belantamab mafodotin (GSK2857916) in relapsed or refractory multiple myeloma based on data from the nominative ATU in France: IFM 2020‐04 study [20]	106	5 (3–12) 66 (37–82)	38.1 3.2 9	37.5 (overall)

Abbreviations: mDOR, median duration of response; mPFS, median progression‐free survival; ORR, overall response rate; PI, proteasome inhibitors.

## 2 PATIENTS AND METHODS

This retrospective study was conducted in 34 Italian centers and included all RRMM patients who received at least one dose of belantamab mafodotin within compassionate use programs named as patient program and expanded access program in Italy. The study was conducted under the aegis of European Myeloma Network Italy. Eligibility criteria included age ≥18 years, MM diagnosis according to international myeloma working group (IMWG) criteria [21], at least four prior LOTs and triple‐refractoriness. Primary endpoint was clinical benefit rate defined as the achievement of at least minimal response (MR) according to IMWG criteria; secondary endpoints were safety, ORR (at least partial response [PR]), duration of response (DoR), PFS, and OS. Triple‐class refractory was defined as being refractory to one IMiD, one PI, and one CD38 mAb and penta‐refractory as refractory to two IMiDs, two PIs, and one CD38 mAb (Moreau ESMO [22]). High‐risk cytogenetics by fluorescence in situ hybridization (FISH) was defined as the presence of t(4;14), t(14;16), del 17p, 1q21 gain or amplification. Non‐ocular toxicity was assessed according to common terminology criteria for AEs version 5.0 (CTCAE v5.0). Ocular toxicity was assessed by the keratopathy and visual acuity scale [23]. Patients had a baseline ophthalmic examination monthly in the first 3 months and on demand subsequently.

The study was conducted in accordance with the Declaration of Helsinki and all patients signed informed consent.

## 3 STATISTICAL METHODS

Data were extracted by review of medical charts and collected using the REDCap electronic data capture tool hosted at Azienda Ospedaliero‐Universitaria Città della Salute e della Scienza di Torino, Torino, Italy.

Patients’ characteristics were summarized by number of observations and percentage for categorical variables or by means of quantiles for continuous variables. Non‐parametric tests were performed for comparisons between groups (chi‐squared and Fisher exact test in case of categorical variables or response rate, Mann–Whitney and Kruskal–Wallis test in case of continuous variables). Survival distributions (e.g., OS, PFS, and DoR) were estimated using the Kaplan–Meier product limit estimator, subgroup comparisons were evaluated by means of the log‐rank test, after assessment of proportionality of hazards. Regarding PFS and OS by response, time was landmarked at the point of measurement of response. All tests were two‐sided, accepting *p* < 0.05 as statistically significant and confidence intervals were calculated at 95% level.

All analyses were performed using the R software (R Core Team [2023]. R: A language and environment for statistical computing. R Foundation for Statistical Computing, Vienna, Austria. URL https://www.R‐project.org/).

## 4 RESULTS

### 4.1 Baseline characteristics

Overall, 78 RRMM patients received at least one dose of belantamab mafodotin between February 2020 and November 2022 and were included in this study. A total of 49% of patients were male, median age at diagnosis was 65 years (range 42–86 years), 51% of patients were older than 65 years and 18% were 75 years of age or older. International Staging System stage II/III was documented in 68% of patients, 9% of them had a creatinine clearance less than 40 mL/min and 7.5% had extramedullary disease. Among 10 evaluable patients at study entry, four (40%) had high‐risk cytogenetics. Eastern cooperative oncology group (ECOG) performance status (PS) was ≥1 in 45% of patients. Median number of prior LOTs was five (range 4–12), 77 patients (99%) had received at least four prior LOTs and 50 patients (64%) at least five prior LOTs; 61 (78%) patients had undergone autologous stem cell transplantation (ASCT) and two patients (2.6%) allogeneic stem cell transplant (Table 2). The median number of belantamab mafodotin cycles administered was two (range 1–40). Baseline characteristics of enrolled patients are detailed in Table 2.

**TABLE 2:** General characteristics of the population.

*N* = 78 (%)			
Sex	Male		38 (49)
	Female		40 (51)
ECOG	0		43 (55)
	1–2		35 (45)
Plasmacytoma	Yes		5 (7.5)
	No		62 (92.5)
Type of MM	IgG		44 (59)
	IgA		21 (28)
	FLC		9 (13)
	Unknown		4
FISH risk	High		4 (40)
	Standard		6 (60)
	Unknown		68
ISS	I–II		30 (59)
	III		21 (41)
	Unknown		27
PLT at baseline		median 138 (50–205)	
	<100		26 (33)
	≥100		52 (67)
Hb at baseline		median 9.8 (7–13.8)	
Previous LOTs		median 5 (4–12)	
	<4		1 (1)
	≥4		77 (99)
Age median		65 (42–86)	
	<70		52 (67)
	≥70		26 (33)
Creatinine clearance at baseline		median 75 (30–74)	
	<40		7 (9)
	≥40		71 (91)

Abbreviations: FISH, fluorescence in situ hybridization; ISS, International Staging System; LOTs, lines of therapy; MM, multiple myeloma; PLT, platelet.

### 4.2 Response, efficacy, and safety

A clinical benefit was obtained by 36 out of 74 evaluable patients (49%), with 43% achieving at least PR, 28% at least VGPR, and 13.5% at least complete response (CR). A total of 20% percent of patients had stable disease and 31% had progressive disease (Table 3). In a subgroup analysis by age, at least MR was achieved by 46% of patients younger than 70 years versus 54% of patients 70 years of age or older (*p* = 0.5); the respective at least PR rate was 42% versus 46% (*p* = 0.8). The two age groups of patients were matched for the main baseline characteristics, except for creatinine clearance, which was significantly lower in the older group, and percentage of patients who had previously undergone ASCT was significantly higher in the younger one.

**TABLE 3:** Response rates of population.

Response	*N* = 78 (%)
Clinical benefit rate (≥MR)	36 (49)
ORR (≥PR)	32 (43)
sCR/CR	10 (13.5)
VGPR	11 (15)
PR	11 (15)
SD	15 (19)
PD	23 (31)
NE	4

Minimal response (MR)	
MR+	36 (49)
MR−	38 (51)
Unknown	4

Minimal response (MR) by subgroups		
	MR+	MR−
ECOG 0	23 (64)	19 (50)
ECOG 1–2	13 (36)	19 (50)
Creatinine clearance < 40	4 (11)	3 (8)
Creatinine clearance ≥ 40	32 (89)	35 (92)
Age < 70	23 (64)	27 (71)
Age ≥ 70	13 (36)	11 (29)

Abbreviations: CR, complete response; MR, minimal response; NE, not evaluable; ORR, overall response rate; PD, progressive disease; PR, partial response; sCR, stringent CR; VGPR, very good partial response.

**FIGURE 1:**
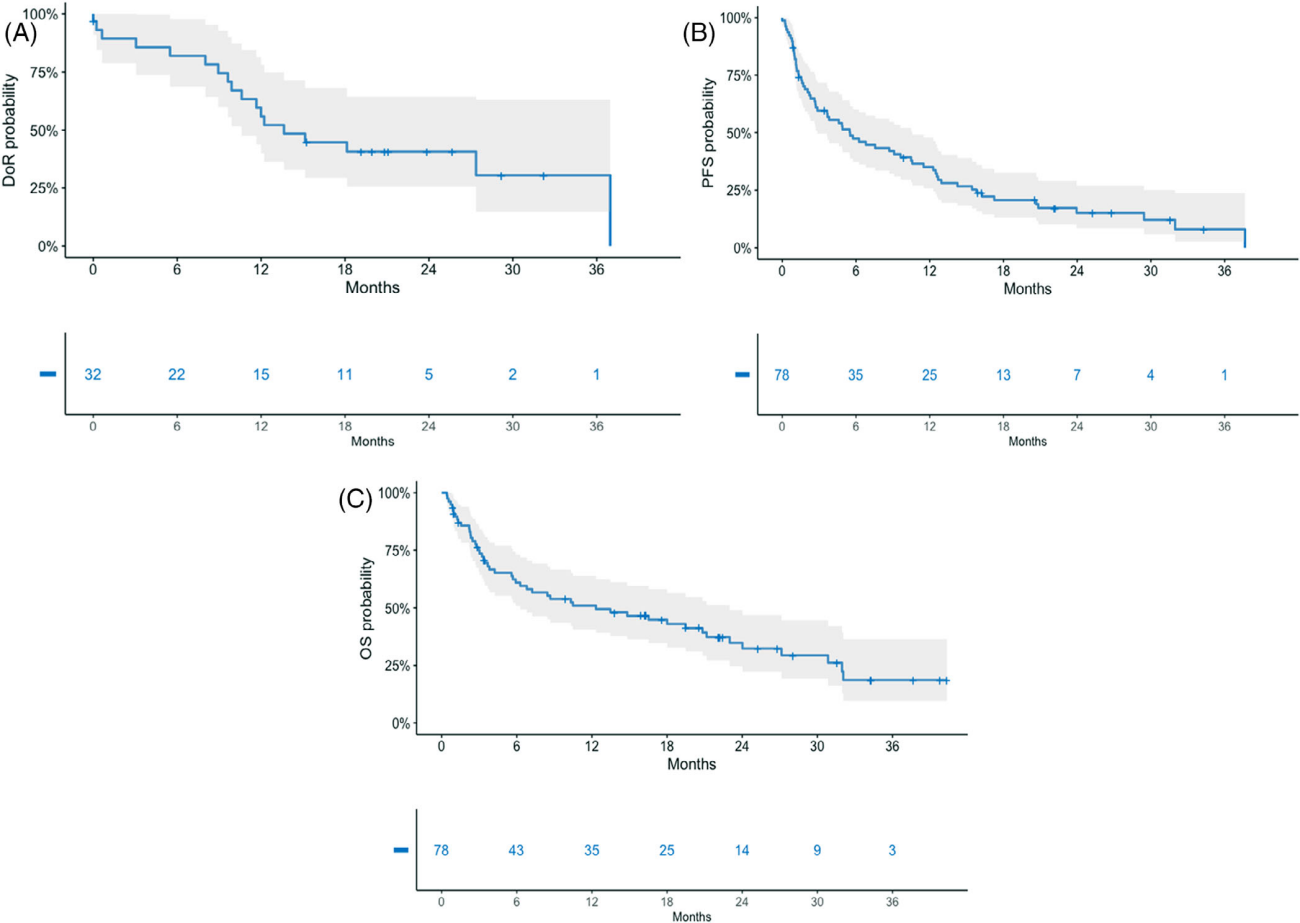
(A) Duration of response (DoR); (B) progression‐free survival (PFS); (C) overall survival (OS) in the population.

Overall, after a median follow‐up of 12 months (range 6–21 months), median DoR was 14 months, with 6‐month DoR of 82% and 12‐month DoR of 56% (Figure 1A). Median PFS was 5.5 months, being 47.5% at 6 months and 35% at 12 months (Figure 1B). Median OS was 12 months (Figure 1C). In the subgroup analysis by age, PFS was 4.9 months in patients younger than 70 years and 6.8 months in those 70 years or older (*p* = 0.32), and it was 13 months in patients aged between 70 and 74 years (Figure S1A). As per ECOG PS, median PFS was 8.7 months in patients with PS 0 and 3.6 months in patients with PS 1–2 (*p* = 0.055) (Figure S2A). Patients with extramedullary disease (EMD) had a median PFS of 1.8 months compared to 8.4 in those without (*p* = 0.001). PFS was significantly longer in responders (achieving at least MR) if compared with non‐responders (median 16 months vs. 2.7 months, *p* < 0.0001) (Figure S3A). Median OS was 12 months in patients younger than 70 years versus 18 months in those 70 years or older (*p* = 0.84) (Figure S1B), and it was 21 months in patients with ECOG PS 0 versus 7.2 months in those with PS 1–2 (*p* = 0.15) (Figure S2B). Median OS was significantly better in patients who responded to belantamab as compared with non‐responders: 27 months versus 4.4 months (*p* < 0.0001) (Figure S3B).

As expected, ocular events represented the most common toxicities. All grade keratopahy occurred in 45 patients (58%), mainly grade ≤2 (55%), with only two patients (2.5%) developing grade 3 keratopathy. Corneal symptoms as blurred vision, dry eye, eye burning, and photophobia were reported in 25 patients (32%), mostly grade 1 (17%) and grade 2 (14%). Only one patient developed grade 3 symptoms. A grade 1–2 reduction in visual acuity was reported in 11 patients (14%). Among 62 patients who underwent ophthalmic assessment before treatment with belantamab mafodotin, 10 (16%) had experienced ocular diseases requiring medical treatment or surgery, five (8%) had a previous diagnosis of cataract, three (5%) had previously suffered from dry eyes, and two patients (3%) had a personal history of glaucoma. A total of 15 (19%) patients temporarily discontinued therapy due to AEs, particularly seven (9%) patients discontinued belantamab for ocular AEs but only two permanently discontinued the drug due to ocular AEs (3%). Ten patients reduced dose of belantamab (13%), with a median dose intensity of 90% (range 77%‐f‐100%).

Thrombocytopenia was the most frequent hematologic toxicity occurring in 16 patients (20.5%), with grade 2 events in nine (11.5%), and grade 3 events in and seven (9%) patients. Four (5%) and five (5.5%) patients developed grade 2 and 3 neutropenia, respectively. Other toxicities included grade 3 pneumonia in one patient (1%) and grade 2–3 diarrhea in two patients (2.6%). Only 3% of patients discontinued belantamab because of side effects. Adverse events are summarized in Table 4 and data about ophthalmic screening before belantamab are shown in Table S4.

**TABLE 4:** Safety in population.

Adverse events	*n* (%)
*Ocular*	*n = 78*
Grade 1	45 (58)
Grade 2	33 (42)
Grade 3	3 (4)
Keratopathy	45 (58)
Symptoms	25 (32)
Blurred vision	17 (22)
Change in BCVA	11 (14)
*Hematological*	*n = 78*
Neutropenia	9 (11.5)
Grade 2	4 (5)
Grade 3	5 (6)
Thrombocytopenia	16 (20.5)
Grade 2	13 (11.5)
Grade 3	7 (9)
Others	
Pneumonia	1 (1)
Diarrhea	2 (2.6)
Discontinuations	
Progression of disease	48 (73)
Death	12 (18)
Adverse event	2 (3)
Lost to follow‐up	1 (1.5)
Withdrawal of consent	0
Screening failure	0
Other	3 (4.5)
Unknown	12

   

## 5 DISCUSSION

Triple‐class refractory MM patients have a poor outcome, and the use of many drug combinations upfront increases the relevance of patient subgroup. The EMA recent approval of novel immunotherapies targeting BCMA, such as bispecific antibody teclistamab and CAR‐T cells idecabtagene‐vicleucel for RRMM patients after at least three LOTs represents an appealing step forward in this setting. However, in many countries, these therapies are not available or are not yet refundable (for instance in Italy), consequently limiting therapeutic options for this population.

In addition, even when CAR‐T cells and bispecific antibodies will be available and refundable, these treatments will likely be administered to fewer patients than those who would in fact benefit from them. Advanced age and poor clinical conditions will be the most important limiting factors. A recent study showed that, due to poor outcome of triple‐refractory MM, only 25% received CAR‐T cell therapy because the median time on the waiting list was 6 months [24]. It should be also emphasized that for CAR‐T cells there is a manufacturing failure between 5% and 20% [25, 26]. Despite the advantage of being “off‐the‐shelf” and immediately available, high costs and the need for at least 1 week hospitalization for side effects monitoring could limit the widespread use of bispecific antibodies compared with ADCs. The ADC belantamab mafodotin has been the first anti‐BCMA immunotherapy licensed for very advanced LOTs in MM patients. This was based on the DREAMM‐2 study showing a deep and durable response in triple‐class refractory RRMM patients who had received a median of seven prior LOTs. Patients receiving belantamab mafodotin 2.5 mg/kg maintained their quality of life (HRQOL) while on treatment and, although daily activities, such as reading and driving, were limited during ocular toxicity episodes, these events did not negatively impact HRQOL and functioning. Indeed, they were temporary and only 3% of patients discontinued belantamab mafodotin because of corneal events [27].

Despite the limitations of cross‐trial comparisons, results of our real‐life series are in line with those of 2.5 mg/kg cohort of DREAMM‐2 study. Patients in both studies were triple‐refractory, and we observed better ORR (43% vs. 32%) and longer PFS (5.5 vs. 2.8 months, respectively) in our study versus DREAMM‐2 study. This was probably due to the lower number of prior LOTs of patients enrolled in our series (median: five lines vs. seven lines in DREAMM‐2). Median OS was similar in both studies: 12 months in our series versus 15.3 months reported in the final analysis of DREAMM‐2 study [3]. Several real‐world studies with belantamab mafodotin monotherapy in RRMM have been recently published, confirming data from DREAMM‐2 trial. Among US real‐life experiences, a trial by the Mayo Clinic including 36 patients with a median of eight prior LOTs showed a median PFS of 2 months and a median OS of 6.5 months, attributed to the poor outcome of patients (19%) who received belantamab after CAR‐T cell therapy. Other retrospective analyses from MD Anderson Cancer Center [7] and Memorial Sloan Kettering Cancer Center [14], in which median number of prior LOTs was seven and six, respectively, showed results comparable with those of DREAMM‐2 study. In the Israeli study [16] including 106 patients with a median age higher than that of our series (69 vs. 65 years), six prior LOTs and 73% triple‐refractory RRMM patients (vs. five prior lines but 100% triple‐refractory in our series), ORR was 45.5% and median PFS 4.7 months. Such data exceed those reported in DREAMM‐2 but are similar to ours. Notably, considering only triple‐refractory RRMM patients of Israeli study, ORR was 43% and median PFS 5.3 months, superimposable to our results. Among European studies [6, 11, 18, 20, 28], the Spanish [18] and French [20] teams conducted and published the two largest studies in RRMM patients treated with belantamab mafodotin. In the multicenter Spanish study, 156 patients received belantamab between 2019 and 2021, median age was 70 years, median prior LOTs were five, 8% of patients were triple‐ and 34.6% penta‐refractory, but no patients received prior anti‐BCMA immunotherapy. In this older and heavily pretreated population, ORR was 41.8%, median PFS 3.6 months and median OS 11.5 months. These results are impressive considering clinical baseline characteristics as creatinine clearance <30 mL/min in 12% of patients, ECOG PS ≥ 1 in 67%, extramedullary disease in 31.4% of them. Remarkably, patients who achieved at least MR had a median PFS of 14.4 months and a median OS of 23.3 months. In the French IFM 2020‐04 real‐world study on belantamab conducted between 2019 and 2020 [20], baseline features of 106 patients were similar to our series, with a median age of 66 years and a median number of prior LOTs of five. However, although all patients were triple‐exposed, only 55.6% were triple‐refractory. Results were surprisingly worse than ours in terms of PFS (median 3.5 months vs. 5 months, respectively) and OS (median 9.3 months vs. 12 months, respectively). We can hypothesize that, over time and thanks to the increased experience with belantamab mafodotin, from the French study to our study, the management of RRMM patients receiving this drug has considerably improved, suggesting the possibility of a learning curve in the use of belantamab mafodotin.

In our study, outcome measures were affected by age, ECOG PS, and response to belantamab. We found no differences in terms of PFS and OS between patients younger than 70 years and 70 years or older. These results are comparable with those reported in the Spanish study [18]: median PFS was 2.6 months in patients younger than 70 years and 3.6 months in those 70 years or older, and median OS was 10 and 12 months, respectively. However, in our study, median PFS and OS in the older population were better than those observed in the Spanish study, and longer than that reported in the DREAMM‐2 trial for the whole study population receiving belantamab 2.5 mg/kg. This suggests that belantamab mafodotin could be a valid therapeutic choice in elderly patients with several comorbidities and who may not be candidates for the most innovative immunotherapies. Another retrospective analysis of 137 patients from the US electronic health record (EHR)‐derived Flatiron Health Database [14], in older patients (median age of 68 years) with cardiovascular and renal comorbidities the ORR was 30.2% and median PFS was 5.4 months.

In addition to age, also ECOG PS—included in several frailty scores—had an impact on PFS and OS in our study. In patients with ECOG PS 0, median PFS and OS were 8.7 months and 21 months, respectively; in patients with ECOG PS 1–2, they were 3.6 months and 7.2 months. However, median PFS of patients with the worst ECOG PS in our series was still better than one reported in the DREAMM‐2 trial for the whole population.

Patients responding to belantamab experienced significantly longer PFS and OS, confirming data from the other real‐life series [16, 18, 20]. Notably, our results are completely overlapping with those of the Spanish study [18]: median PFS was 14.4 months in patients achieving at least MR versus 1.6 in non‐responders; and median OS was 23.3 months versus 3.2 months, respectively. As expected, ocular toxicities—mainly keratopathy—represented the most frequent side effects, occurring in 58% of patients. However, although about 25% of patients had prior eye diseases, keratopathy was mainly of grade 1–2 (55%) and only 3% of patients discontinued treatment due to belantamab toxicity. Improved experience with belantamab over time and periodic ophthalmologic evaluations have limited the occurrence of most serious ocular events.

The retrospective design is the main limitation of our analysis, leading to some missing data for some patients. However, in this multicenter, real‐life study, in which no exclusion criteria were established, belantamab mafodotin has shown to induce significant and durable responses in triple‐refractory RRMM patients. Particularly interesting are the results obtained in the older population, where the benefit of belantamab seems to be more evident than in younger patients. Our data confirm that belantamab mafodotin is a valid option in a patient population that could not be a candidate for CAR‐T cell therapy or bispecific antibodies.

## AUTHOR CONTRIBUTIONS

Maria Teresa Petrucci, Mario Boccadoro, Massimo Offidani, and Sonia Morè designed the study; Laura Corvatta, Massimo Offidani, and Sonia Morè wrote the manuscript; Alfonso Piciocchi performed statistical analyses and made the figures; Francesca Fazio, Maria Teresa Petrucci, Roberta Della Pepa, EZ, Maurizio Musso, Renato Zambello, Angelo Belotti, Sara Bringhen, Elisabetta Antonioli, Concetta Conticello, Nicola Di Renzo, Valerio De Stefano, Pellegrino Musto, Barbara Gamberi, Daniele Derudas, Massimo Offidani, and Sonia Morè were responsible for the recruitment, follow‐up, management of patients and acquired the data; all authors contributed to the interpretation of the results and approved the final manuscript.

## CONFLICT OF INTEREST STATEMENT

Francesca Fazio ‐ advisory board: GSK; honoraria: Amgen, Takeda, Janssen‐Cilag, GSK, BeiGENE, and Sanofi

Maria Teresa Petrucci ‐ honoraria: Janssen‐Cilag, Celgene‐BMS, Amgen, Sanofi, GSK, and Takeda; advisory boards: Janssen‐Cilag, Celgene‐BMS, Amgen, Sanofi, GSK, Takeda, Roche, Oncopeptides, Pfizer, Menarini, and AbbVie; support for attending meetings and/or travel: Janssen‐Cilag, Celgene‐BMS, Amgen, Sanofi, and Takeda.

Laura Corvatta ‐ honoraria: BMS, Janssen, and GSK.

Alfonso Piciocchi ‐ no conflicts of interest.

Roberta Della Pepa ‐ advisory boards: Amgen, Bristol Myers Squibb, Celgene, GlaxoSmithKline, Janssen, Sanofi, and Takeda.

Paola Tacchetti ‐ honoraria: Amgen, Bristol‐Myers Squibb/Celgene, Janssen, Takeda, AbbVie, Sanofi, GlaxoSmithKline, and Pfizer.

Maurizio Musso ‐ no COI.

Renato Zambello ‐ advisory boards: Roche, Janssen, Bristol Meier Squibb, Sanofi, Amgen, and GSK.

Angelo Belotti ‐ advisory boards: Amgen, GSK, Janssen, Takeda, and Pfizer.

Sara Bringhen ‐ speakers’ bureaus: Amgen, Bristol Myers Squibb, GlaxoSmithKline, Janssen, Sanofi, and AbbVie; advisory boards: Bristol Myers Squibb, Janssen, Takeda, Pfizer, Stemline Therapeutics, and Oncopeptides; consultancy fees: Sanofi.

Elisabetta Antonioli ‐ advisory boards: Janssen‐Cilag, Celgene‐BMS, Amgen, Sanofi, GSK, Takeda, and Pfizer; support for attending meetings and/or travel: Janssen‐Cilag and Sanofi.

Concetta Conticello ‐ honoraria: Takeda, Amgen, Janssen, GSK, BMS, and Sanofi.

Nicola Di Renzo ‐ honoraria: Janssen, Bristol Myers Squibb, Gilead, Jazz, and AbbVie; advisory boards: Janssen, Bristol Myers Squibb, Jazz, and AbbVie.

Valerio De Stefano ‐ advisory boards for: AOP Health, Argenx, Bristol Myers Squibb, Glaxo Smith Kline, Grifols, Novartis, SOBI, and Takeda; speaker fees from Abbvie, Alexion, Amgen, Bristol Myers Squibb, Grifols, Leo Pharma, Novartis, Novo Nordisk, Sanofi, and Takeda; research grant from Alexion.

Pellegrino Musto ‐ honoraria: Abbvie, Alexion, Amgen, Astellas, Astra‐Zeneca, Bei‐Gene, Bristol‐Myers Squibb/Celgene, Gilead, Glaxo‐Smith‐Kline, Grifols, Incyte, Janssen, Jazz, Novartis, Pfizer, Roche, Sanofi, Sobi, and Takeda.

Barbara Gamberi ‐ honoraria: Amgen, Bristol Myers Squibb, Janssen, and Takeda; advisory boards: Amgen, Bristol Myers Squibb, GlaxoSmithKline, Janssen, Sanofi, and Takeda.

Daniele Derudas ‐ no conflicts of interest.

Mario Boccadoro ‐ honoraria: Sanofi, Celgene, Amgen, Janssen, Novartis, Bristol Myers Squibb, and AbbVie; advisory boards: Janssen and GlaxoSmithKline; research funding: Sanofi, Celgene, Amgen, Janssen, Novartis, Bristol Myers Squibb, and Mundipharma.

Massimo Offidani ‐ honoraria and advisory boards: Amgen, Bristol Myers Squibb, Celgene, GlaxoSmithKline, Janssen, Sanofi, and Takeda.

Sonia Morè ‐ honoraria: BMS, Janssen, and GSK.

## FUNDING INFORMATION

This study is funded from EMN (European Myeloma Network).

## ETHICS STATEMENT

The authors have confirmed ethical approval statement is not needed for this submission.

## PATIENT CONSENT STATEMENT

The authors have confirmed patient consent statement is not needed for this submission.

## CLINICAL TRIAL REGISTRATION

The authors have confirmed clinical trial registration is not needed for this submission.

## Supporting information

Supporting Information

Supporting Information

## Data Availability

The data that support the findings of this study are available on request from the author Alfonso Piciocchi.
