# Supplementary material for: Belantamab mafodotin in triple‐refractory multiple myeloma patients: A retro‐prospective observational study in Italy
Source: EJHaem. 2024 Apr 30;5(3):485–93. doi: 10.1002/jha2.907 (PMC11182418; doi:10.1002/jha2.907)
Supplement: Supplementary file 1 — Supporting Information [file JHA2-5-485-s001.docx]

Figures S1. PFS and OS according age (<70 *vs* ≥70 age)

**A**


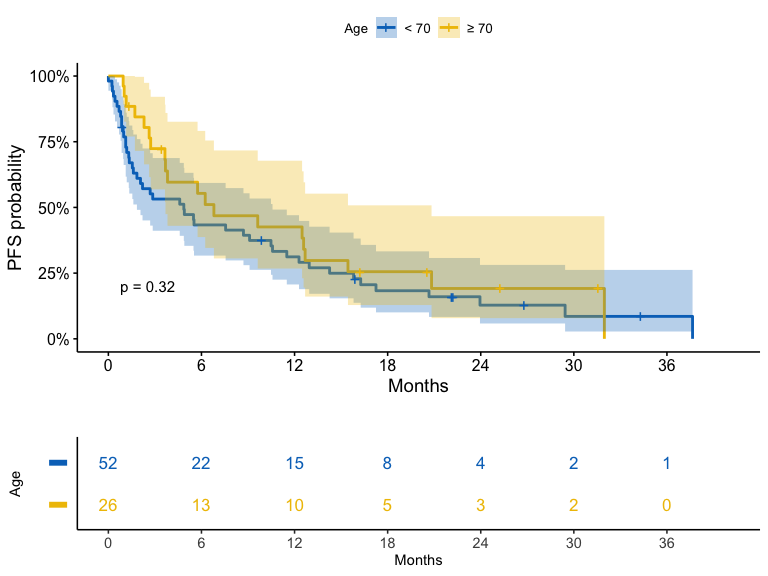


**B**

Figures S2. PFS and OS according performance status (ECOG 0 *vs* ≥ 1-2)

**A**


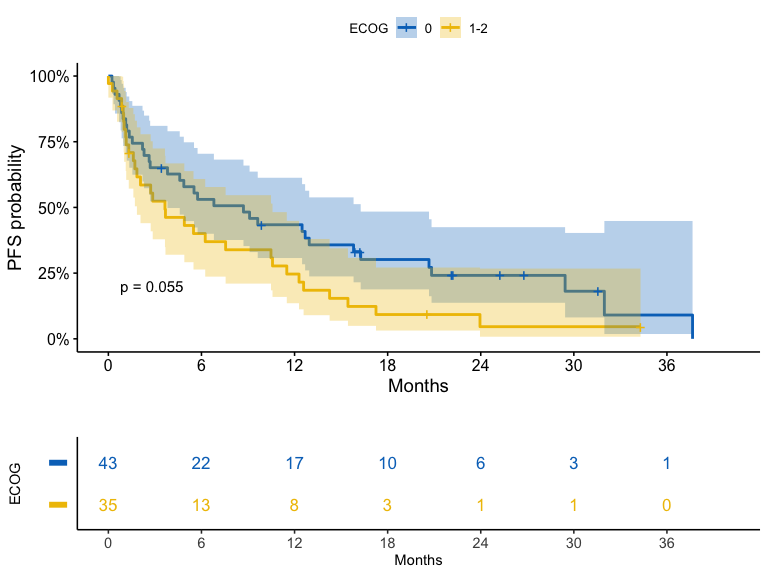


**B**


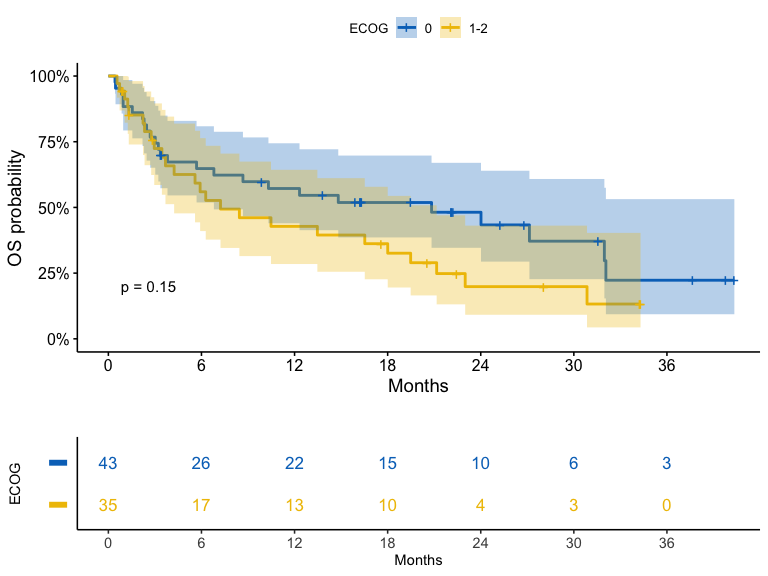


Figures S3. PFS and OS according disease response (≥ MR *vs* < MR)

**A**


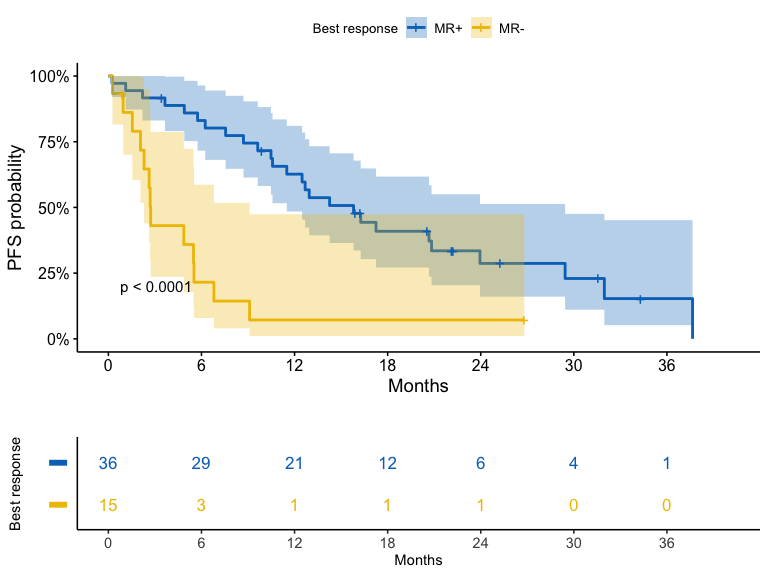


**B**


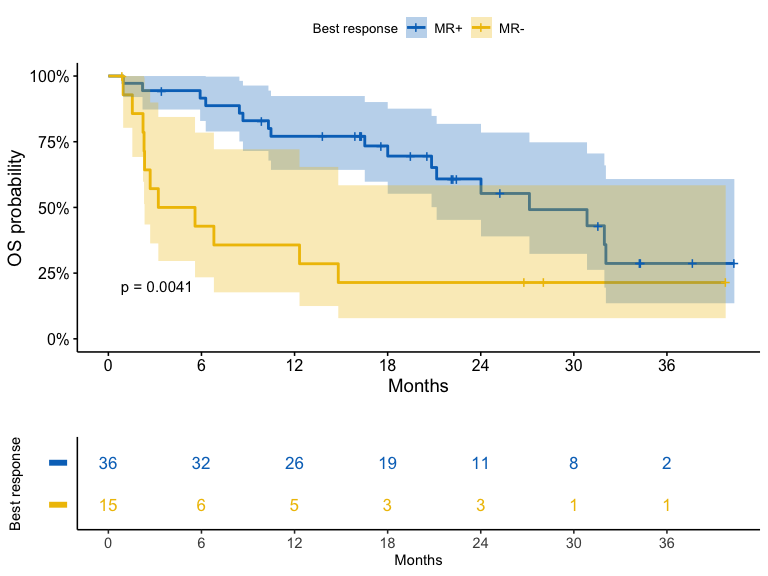


| **Characteristics** | **N=78 (%)** | | |
| --- | --- | --- | --- |
| **Ophthalmic screening at baseline** | Yes | 62 (81) |  |
|  | Unknown | 1 |  |
| **Chronic topical steroid use** | Yes | 0 |  |
|  | Unknown | 22 |  |
| **History of intraocular surgery and/or laser treatment surgery** | Yes | 1 (2) |  |
|  | Unknown | 21 |  |
| **History of ocular disease requiring medical treatment** | Yes | 5 (9) |  |
|  | Unknown | 22 |  |
| **Serious eye trauma** | Yes | 0 |  |
|  | Unknown | 21 |  |
| **Previous diagnosis of dry eye** | Yes | 3 (5) |  |
|  | Unknown | 21 |  |
| **Personal history of glaucoma** | Yes | 2 (3.5) |  |
|  | Unknown | 21 |  |
| **Previous diagnosis of cataract** | Yes | 5 (9) |  |
|  | Unknown | 20 |  |

Figures S4. Ophthalmic screening at baseline
