# Supplementary material for: Belantamab mafodotin in triple‐refractory multiple myeloma patients: A retro‐prospective observational study in Italy
Source: EJHaem. 2024 Apr 30;5(3):485–93. doi: 10.1002/jha2.907 (PMC11182418; doi:10.1002/jha2.907)
Supplement: Supplementary file 2 — Supporting Information [file JHA2-5-485-s002.docx]

**Appendix**

**Belantamab mafodotin in triple-refractory multiple myeloma patients: a retro-prospective observational study in Italy**

**We are grateful to all participating sites:**

Ospedale S Giovanni Addolorata – Roma (V. Bongarzoni)

ASST Dei Sette Laghi – Varese (M. Brociner)

AUSL della Romagna – Ravenna (C. Cellini)

CRO Aviano – Aviano (R. Ciancia)

ASST Ovest Milanese - Legnano (A. Corso)

Fondazione IRCCS Ca' Granda - Ospedale Maggiore Policlinico – Milano (M. C. Da Vià)

AO Maggiore della Carità – Ferrara (L. De Paoli)

Fondazione PTV Policlinico Tor Vergata - Roma (L. Franceschini)

ASST Fatebenefratelli Sacco – Milano (S. Girlanda)

Istituto Oncologico Veneto Ospedale Busonera - Castelfranco Veneto (M. Gottardi)

Azienda Ospedaliera Universitaria Senese – Siena (A. Gozzetti)

Azienda USL Toscana Centro - Prato (S. Grammatico)

AO S. Luigi Gonzaga - Orbassano (T. Guglielmelli)

Ospedale Perrino – Brindisi (G. Mele)

Pia Fondazione Panico – Tricase (M. Mele)

Ospedale Moscati - ASL Taranto (G. Palazzo)

Istituto Tumori Giovanni Paolo II – Bari (A. M. Quinto)

AO Universitaria di Ferrara - Ospedale di Cona (M. Sessa)

Azienda USL di Pescara (F. Fioritoni)
